# The Railway Surgeons and the Law

**Published:** 1894-05

**Authors:** Clark Bell

**Affiliations:** Of the New York Bar, President Medico-Legal Society, Editor Medico-Legal Journal


### TEXAS MEDICAL JOURNAL.

ESTABLISHED JULY, 1885.

PUBLISHED MONTHLY.—SUBSCRIPTION $2.00 A YEAR.

VOL. IX. AUSTIN, MAY, 1894. NO. 11.

### Original Contributions.

For Texas Medical Journal.

### THE RAILWAY SURGEON AND THE LAW.

BY CLARK BELL, ESQ., OF THE NEW YORK BAR,
(President Medico-Legal Society, Editor Medico-Legal Journal.)

Read before the Medico-Legal Society of New York, April, 1894. Read before the Erie Railway Surgeons January, 1894. Published from advanced sheets of the Medico-Legal Journal.

THE railway is the most important factor in its influence upon civilization, that the present century has produced.

The first railway was operated as early as 1829, but it was in 1832, and later, that the packet boat on the canals, which had superseded the stage coach, gave way to the locomotive.

The recent Columbian Exposition at Chicago illustrated practically the growth and evolution of American railways during the past sixty years. The early locomotives and the improvements stood there side by side.

It has been a gradual, but as a whole, a grand, a magnificent evolution, creditable to the genius of our people, and without which, the stupendous and marvelous development of our country, would have been impossible, and which even with its wonder working changes, is well nigh inexplicable.

Our century has been well called the age of steam and electricity. Railways and the electric telegraph have been the hand-maidens of the genius of civilization which have transformed the wilderness of this portion of the American continent into a lovely land of beautiful homes, of a great people; which excites alike the wonder, the admiration, and the envy of mankind.

With these enormous advantages, which the construction and operation of railways have bestowed, almost as a legacy, upon the race, have also come enormous responsibilities, great antagonisms and the creation of new laws, rules and conditions, which recognizing the magnitude of the blessings conferred, have grappled with the problems of the relation of the railway to the general public, and its responsibility as a common carrier, under those elementary principles of law that had been created for the era, and methods in use before the transformations due to electricity and steam.

Necessity and practical trial, has been the stern, the inexorable and the costly teacher, to the managers and promoters of railways, and the lessons taught in such a school furnish that knowledge gained by experience, which all will agree is the most valuable of all human knowledge, and usually the most costly. The railway surgeon, like the railway lawyer, became a necessity of the situation.

It took a longer time and a broader comprehension of the true field of proper administration and economic management, to illustrate to railway officials the absolute necessity of a competent and thorough surgical service, than it did of the necessity of legal counsel. The one had to be engaged from the organization, and during the preliminary steps of construction, while the other came after the railway was completed, and when the inevitable and sometimes fortunately, long deferred accident occurred.

Surgery itself which had been stagnant and torpid during more than the first half of the struggle, began to show signs of awakening, and during the last twenty five years, has developed a growth as marvelous, in many respects, as that of which we have spoken, and which has excited the interest, the admiration and the wonder of the age.

Whatever may be said of medicine, and its practice as a science, surgery is now recognized everywhere as an exact science, and its evolution and progress in the past twenty years of the present century has been greater and more conspicuous than during all the intervening years of the Christian era.

It has not been, and never can be, a question of schools, as in medicine.

Surgery rests on firm, secure, solid and sound foundations. Electricity is at this moment the splendid trained obedient servant of surgery, and some of its grandest and proudest recent achievements are due to this wonderful and mysterious agent.

The managers of railways, as a class, are of the ablest, most comprehensive, and most skillful men in our land.

They who have thought most and best upon this subject have utilized the surgeon as one of the most valuable helpers to the railway in its relations to the community which is the patron of the railways.

The great railways now nearly all have each its chief surgeon, and its local surgeons; those who do not, are behind the age, and not in touch with the progress of events, or the needs of the hour.

THE RELATION OF THE SURGEON TO THE LAW.

I cannot, in the space of a short paper, speak in detail upon so important a question, and shall only give outlines or suggestions at this time.

It goes without saying that the surgeon should be profoundly versed in the especial knowledge of his own profession. He should not hesitate for a moment, to place himself *en rapport*, and abreast of the advance of surgical scientific thought, knowledge, and discovery.

The ablest surgeon would find great advantage by short courses of study at the great centres where practical and experimental work, illustrating the latest and most advanced discussions, can be seen and studied.

His education should embrace also a knowledge of the science of forensic medicine, to enable him to understand the questions of responsibility in their elementary bearings, in what are commonly know as railway damage cases.

I do not mean that a railway surgeon should aim to become a railway lawyer, but I do insist that he should not consent to be ignorant of the elementary principles of law involved; nor should he be ignorant of the well settled and well established principles and decisions which should govern such cases in the courts.

As his professional duties call him to the stand as a medical expert witness, he is bound by every consideration to qualify himself for the proper discharge of that duty.

It is not enough that he understand his own profession alone; it is part of his duty, whether for the company, or for the injured, to know how to give his evidence, what his rights are on the stand as a witness, what is proper for him in his position to state, and properly to state it, and what he should not state. Many a physician who has neglected this field of study, has lost his reputation, and destroyed his prospects—sometimes in an hour,—by inexcusable blunders on the stand as a witness.

It would require an essay to define this in detail. A knowledge of the law of the powers and duties of expert witnesses, and of expert and opinion evidence, is almost as necessary for the expert witness when a railway surgeon, as for the counsel who tries the case, and a railway surgeon, who recognizes this and provides for it, is wise, and if he neglects, he is not wise.

RAILWAY COMPANIES AND THEIR INTEREST IN GOOD SURGEONS.

No class of litigants are exposed to such dangers in damage cases as railway corporations.

Without knowing why, all counsel recognize the fact that the sympathies of jurors are not only against the corporations, but this sympathy often seems to have higher claims and stronger help upon the average juryman, than conscience or the sense of abstract justice.

A different code of moral ethics seems to exist between corporations and poor claimants than between man and man, in the estimation of juries.

It is not an overestimate to place the losses of railways, in damage cases, by miscarriage of justice, at millions of dollars. With some considerable thought and reflection upon this subject, I am of the opinion that, in many of these cases, a competent railway surgeon could have saved his company large sums of these losses.

Take the case, for example, cited by that eminent Nestor of American Surgeons, Prof. Lewis A. Sayre, in his paper read before the Erie Railway Surgeons in January last.

Dr. C. W. Hackett, in 1877, met with a railway accident on the Sangus branch of the Eastern railroad, between Boston and Maplewood, at Everett Junction, that produced apparently incurable paralysis. He brought suit and recovered an enormous verdict, some $39,000 or $40,000. Prof. Sayre, in 1879, was called in, treated and suspended him; took the pressure off the injured parts, and cured him by his methods of suspension, with plaster of paris jackets.

Had Dr. Sayre been the surgeon of the corporation, or any surgeon who understood his business, the patient would have been saved two years of suffering, and the company, probably, $30,000 in cash, aside from the cost and expenses of the trial.

Intelligent railway management to-day, as a matter of business, duty, and fair dealing, always desire to adjust railway injuries without trials or law suits, for they realize their danger before juries.

The intelligent railway surgeon is as necessary to shield the corporation from an exorbitant, improper or fictitious claim, as he is to aid the injured, in case of a substantial injury, to recover a proper and adequate redress.

The pressure upon my time has been such that I only can submit a few thoughts for your consideration, which must needs be brief and, perhaps, unsatisfactory, but the growing importance of railway surgery, its recognition by surgeons throughout the country, by railway managers, and the interest it has excited in the recent past, emboldens me to call attention to the movement in the Medico-Legal Society, to organize a section upon the medical jurisprudence of surgery, that has beeu called that of railway surgery, which embraces all surgical questions where medico-legal issues arise. Eminent surgeons of the National Association of Railway Surgeons have taken a deep interest in its organization, and have joined that society and united with that section.

The questions here discussed, and, indeed, all the broad fields of railway and medical-legal surgery will be within the scope of its domain of investigation, and it should be the means of enlarging the horizon lines of surgical thought to all students of the medical jurisprudence of surgery, whether of the medical or legal professions, and can not fail to be of interest to railway managers and railway men generally, who are eligible to its membership, whether they are lawyers or surgeons.

An initiation fee of $5 and annual dues of $2, outside the State of New York, entitle each member to the *Medico-Legal Journal* free of charge; and the section already formed is under the management of a chairman and ten vice-chairmen, selected from each profession, from among the most prominent surgeons in the National Association of Railway Surgeons, of which the Surgeon General of the United States has consented to be one, and upon the legal side from the most distinguished railway lawyers and surgeons throughout the United States, who are connected with that body.

The following are the officers of the Section on Railway Surgery of the Medico-Legal Society for 1894:

FOR CHAIRMAN:

Granville P. Conn, M. D., of New Hampshire.

VICE-CHAIRMEN:

*Legal.*

Clark Bell, Esq., of New York.

Hon. C. H. Blackburn, of Ill.

Judge John F. Dillon, of N. Y.

Judge L. A. Emery, of Maine.

Judge W. H. Francis, of Mont.

Hon. Geo. W. Fellows, of N. H.

Hon. W. C. Howell, of Iowa.

Hon. Geo. R. Peck, of Ill.

Hon. J. M. Thurston, of Neb.

*Surgical.*

George M. Sternberg, M. D., Surgeon General of the U. S.

Charles E. Cole, M. D., of Mont.

W. J. Galbraith, M. D., of Neb.

R. S. Harnden, M. D., of N. Y.

J. B. Murdoch, M. D., of Penn.

Thos. H. Manley, M. D., of N. Y.

W. B. Outten, M. D., of Mo.

R. Harvey Reed, M. D., of Ohio.

Nicholas Senn, M. D., of Ill.

S. S. Thorne, M. D., of Ohio.

*Secretary.*

Clark Bell, Esq., of New York.

*Treasurer.*

Geo. Chaffee, M. D., of Brooklyn.

FOR EXECUTIVE COMMITTEE:

Clark Bell, Chairman.

George Chaffee, M. D., of N. Y.

Granville P. Conn, M. D., N. H.

Judge A. H. Dailey, of N. Y.

Judge John F. Dillon, of N. Y.

H. W. Mitchell, M. D., of N. Y.

W. B. Outten, M. D., of Mo.

R. Harvey Reed, M. D., of Ohio.

Chief Surg. B. F. Eads, Texas.

Chief Surgeon James M. Dinnen, of Indiana.

